# Development of Human Breast Milk Microbiota-Associated Mice as a Method to Identify Breast Milk Bacteria Capable of Colonizing Gut

**DOI:** 10.3389/fmicb.2017.01242

**Published:** 2017-07-11

**Authors:** Xiaoxin Wang, Huifang Lu, Zhou Feng, Jie Cao, Chao Fang, Xianming Xu, Liping Zhao, Jian Shen

**Affiliations:** ^1^Key Laboratory of Systems Biomedicine (Ministry of Education), Shanghai Center for Systems Biomedicine, Shanghai Jiao Tong University Shanghai, China; ^2^State Key Laboratory of Microbial Metabolism, School of Life Sciences and Biotechnology, Shanghai Jiao Tong University Shanghai, China; ^3^Shanghai General Hospital Shanghai, China

**Keywords:** breast milk, germfree mice, gut, bacteria, gnotobiotic

## Abstract

Human breast milk is recognized as one of multiple important sources of commensal bacteria for infant gut. Previous studies searched for the bacterial strains shared between breast milk and infant feces by isolating bacteria and performing strain-level bacterial genotyping, but only limited number of milk bacteria were identified to colonize infant gut, including bacteria from *Bifidobacterium*, *Staphylococcus*, *Lactobacillus*, and *Escherichia*/*Shigella*. Here, to identify the breast milk bacteria capable of colonizing gut without the interference of bacteria of origins other than the milk or the necessity to analyze infant feces, normal chow-fed germ-free mice were orally inoculated with the breast milk collected from a mother 2 days after vaginal delivery. According to 16S rRNA gene-based denaturant gradient gel electrophoresis and Illumina sequencing, bacteria at >1% abundance in the milk inoculum were only *Streptococcus* (56.0%) and *Staphylococcus* (37.4%), but in the feces of recipient mice were *Streptococcus* (80.3 ± 2.3%), *Corynebacterium* (10.0 ± 2.6 %), *Staphylococcus* (7.6 ± 1.6%), and *Propionibacterium* (2.1 ± 0.5%) that were previously shown as dominant bacterial genera in the meconium of C-section-delivered human babies; the abundance of anaerobic gut-associated bacteria, *Faecalibacterium*, *Prevotella*, *Roseburia*, *Ruminococcus*, and *Bacteroides*, was 0.01–1% in the milk inoculum and 0.003–0.01% in mouse feces; the abundance of *Bifidobacterium* spp. was below the detection limit of Illumina sequencing in the milk but at 0.003–0.01% in mouse feces. The human breast milk microbiota-associated mouse model may be used to identify additional breast milk bacteria that potentially colonize infant gut.

## Introduction

Although human breast milk-associated microbiota is dominated by skin-associated bacteria *Staphylococcus* and *Streptococcus* (Jost et al., 2013; Charbonneau et al., 2016), breast milk is considered an important source of commensal bacteria for the neonatal gut, because DNA of gut-associated bacteria, including *Bacteroides*, *Clostridium*, *Faecalibacterium*, *Roseburia*, and *Bifidobacterium*, etc., have been repeatedly detected at low abundance in milk in different studies using 16S rRNA gene-based molecular approaches (Gueimonde et al., 2007; Martin R. et al., 2007; Perez et al., 2007; Collado et al., 2009; Hunt et al., 2011; Cabrera-Rubio et al., 2012; Jost et al., 2013, 2014). To investigate what breast milk bacteria can colonize infant gut, i.e., the mother-neonate vertical transfer of bacteria via breastfeeding, numerous studies searched for the bacterial species/strains shared between breast milk and infant feces in mother-infant pairs by microbiologically isolating bacteria and strain-level genotyping of the bacterial isolates (Martin et al., 2006, 2012; Jost et al., 2014). From thousands of bacterial isolates of the maternal breast milk and infant feces, only a limited number of bacterial species (*Bifidobacterium breve*, *B. longum*, *Staphylococcus epidermidis*, *S. hominis*, *Lactobacillus fermentum*, *L. gasseri*, *L. plantarum*, *L. reuteri*, *L. salivarius*, *L. vaginalis*, and *Escherichia*/*Shigella* spp.) were found to be shared by a few mother-infant pairs (Martin et al., 2006, 2012; Jost et al., 2014). This is probably because the human neonatal gut microbiota receives bacteria from multiple sources other than breast milk, including mothers’ feces, vaginal tract, skin and the surrounding environment during delivery. Therefore, alternative methods are needed to identify additional candidate bacteria that are potentially transferred from the mothers’ breast milk to infant gut.

Germ-free mice provide an animal model in which the source of commensal bacteria can be strictly controlled and microbiological contamination from other origins is avoided. They have been shown to be an effective surrogate host of human gut bacteria (Kibe et al., 2005; Turnbaugh et al., 2009; Ridaura et al., 2013). Over 85% of the bacterial genera present in the adult human donors’ feces, including *Bacteroides*, *Faecalibacterium*, and *Roseburia*, etc., can be detected in recipient ex-germ-free mice that were gavaged with human fecal suspension (Turnbaugh et al., 2009). A human baby microbiota, which consisted of bacteria of *Bacteroides*, *Enterobacteria*, *Bifidobacterium*, *Lactobacillus*, and *Staphylococcus* isolated from the feces a 20-day-old female baby, can stably colonize the gut of germfree mice (Martin et al., 2007). No previous study has transplanted human breast milk microbiota to germfree mice to screen for the breast milk bacteria that can colonize the gut.

In the present study, germ-free mice fed on normal chow were inoculated orally with the breast milk of one 38-year-old mother 2 days after vaginal delivery, and the microbiota composition of milk inoculum and mouse feces were compared with 16S rRNA gene profiling and microbiological culture techniques.

## Materials and Methods

### Subject and Breast Milk Collection

The breast milk was collected from a 38-year-old mother 2 days after vaginal delivery at term. The mother had gestational diabetes mellitus during pregnancy (the serum glucose levels of Oral Glucose Tolerance Test were fasting 4.23 mmol/L, 1 h 10.6 mmol/L, and 2 h 10.28 mmol/L). She had no gastrointestinal diseases, immunological disorders, infectious diseases, or organic diseases. The mother received no antibiotics within 3 months before breast milk sampling, and she performed exclusive breastfeeding when the milk sample was collected. The protocol of the study was approved by the Ethical Committee of Shanghai General Hospital. Written informed consent was obtained from the mother before the participation in the study.

The breast was first washed with sterile water, subsequently, the nipple and areola were swabbed with An’erdian^®^ type III skin antiseptic solution containing 0.5% (w/v) available iodine and 0.1% (w/v) chlorhexidine gluconate (LiKang, Shanghai, China) and then swabbed with sterile water. Wearing single-use sterile rubber surgical gloves, the nurse manually collected the breast milk into a sterile tube after discarding the first drops (∼100 μl).

The breast milk was immediately transported to the lab in an anaerobic jar. Aliquots of the breast milk were inoculated to germ-free mice, and processed for bacterial cultivation in an anaerobic chamber within 2 h after collection. Further aliquots were stored at -80°C for DNA extraction.

### Animal Experiments

All experimental procedures and protocols were approved by the Institutional Animal Care and Use Committee of Laboratory Animals of Shanghai Laboratory Animal Center (SLAC), Chinese Academy of Sciences, Shanghai, China.

Ten weaned germfree male C57BL/6J mice were raised in a Trexler-type flexible-film plastic isolator with a regular 12 h light cycle (lights on at 0600 h) in SLAC. They were provided with sterile normal chow (containing 4.62% fat, 3.45 kcal g^-1^, from SLAC Inc., Shanghai, China) and water *ad libitum*. Periodic bacteriologic examination of feces with bacterial cultivation was performed to make sure there was no bacterial contamination.

At the age of 8 weeks, each of the 10 mice was inoculated with 100 μl freshly collected breast milk by gavage, and a repeat inoculation of the identical milk sample was conducted on the third day. The experiment lasted 8 weeks since the first inoculation. Fresh stool samples were collected from each mouse weekly and frozen at -80°C for DNA extraction. At week 8, one aliquot of the feces collected from No. 4 recipient mouse was used for bacterial isolation.

### DNA Extraction from the Breast Milk and Mouse Feces

Two milliliters of breast milk was centrifuged at 9,000 × *g* for 20 min to collect the bacterial cell pellets. For the feces of mice, one fecal pellet was homogenized in 0.5 ml phosphate buffered saline supplemented with 0.05% (w/v) L-cysteine and centrifuged as above. Total DNA was extracted from the resultant bacterial cell pellets as previously described (Godon et al., 1997) and as specified in Supplementary Information, and purified with Omega Gel Extraction kit (D2501-01, OMEGA Bio-Tek, Taiwan, China). The integrity of the DNA was assessed by using 0.8% agarose gel electrophoresis gels stained with ethidium bromide, and the concentration was quantified with PicoGreen fluorescent dye (Thermo Fisher Scientific, Sunnyvale, CA, United States) by using SpectraMax M5 microplate reader (Molecular Devices, San Francisco, CA, United States).

### DGGE of 16S rRNA Gene V3 Region Amplicons

The 16S rRNA gene V3 region was PCR amplified with the genomic DNA extracted from the breast milk and feces of recipient mice as the template. The primer p2 (5′-ATTACCGCGGCTGCTGG-3′) and p3 (5′-CGCCCGCCGCGCGCGGCGGGCGGGGCGGGGGCACGGGGGGCCTACGGGAGGCAGCAG-3′) (Muyzer et al., 1993) were used. The 25 μl PCR mixture contained 10 ng of DNA templates, 0.75 U of TaKaRa rTaq polymerase (Takara, Dalian, China), 1× PCR buffer (Mg^2+^ free), 2 mM MgCl_2_, 6.25 pmol of each of the primer, each deoxynucleoside triphosphate at a concentration of 200 μM. The PCR program included the following steps: an initial denaturation at 94°C for 3 min; 20 cycles of touchdown PCR consisting of denaturation at 94°C for 1 min, annealing for 1 min at temperatures decreasing from 65 to 55°C with 1°C interval every second cycle, and extension at 72°C for 1 min; 5 cycles of regular PCR (94°C for 1 min, 55°C for 1 min, and 72°C for 1 min); a final extension step for 6 min at 72°C. The sizes of PCR products were assessed using 1.5% agarose gel electrophoresis gels stained with ethidium bromide.

DGGE was performed with the Dcode System apparatus (Bio-Rad, Hercules, CA, United States). PCR products (300 ng) were separated on 8% (w/v) polyacrylamide gels with a denaturing gradient of 27–55%. The 100% denaturant corresponds to 7 M urea and 40% deionized formamide. Electrophoresis was performed in 1× Tris–acetate–EDTA (TAE) buffer at a constant voltage of 200 V and a temperature of 60°C for 4 h. Gels were stained with SYBR green I (Amresco, Solon, OH, United States) and were photographed with a UVI gel documentation system (Tanon-3500, Beijing, China).

Quantity One software (version 4.4.0, Bio-Rad, Hercules, CA, United States) was used to digitize the DGGE profiles by determining the migration position and intensity of DGGE bands. Bands migrating to an identical position were considered to represent the same bacterial species. Dendrogram of the DGGE profiles was generated based on the similarity of profiles with UPGAMA clustering analysis using the Quantity One software.

### DNA Sequencing of DGGE Bands

The DGGE bands were excised from the gels with a sterile knife and incubated in 100 μl sterile distilled water at 4°C overnight. The 16S rRNA gene V3 region in the band was re-amplified using 4 μl eluate as the template and the primer pair p2 and p3. PCR products were purified using the Gel Extraction Kit (Omega, United States), ligated into the pGEM-T easy vector (Promega, Madison, WI, United States), and transformed into competent *Escherichia coli* DH5a cells. Positive clones were picked randomly, and inserts were amplified and screened for their migration position by DGGE. Clones that migrated to the same position as the original DGGE bands were sequenced (Life Technologies, Shanghai, China).

### Illumina Sequencing of Bacteria 16S rRNA Gene V3–V4 Regions

For the breast milk and feces of recipient mice, the sequencing library of 16S rRNA gene V3–V4 regions was prepared with two steps of amplification according to the protocol provided by Illumina^1^ with the following modifications. For the Amplicon PCR (amplification of 16S rRNA gene V3–V4 region), the 25 μl reaction mix consisted of 1.6× Pfx amplification buffer, 1 mM MgSO_4_, 0.3 mM dNTP, 0.2 μM of each specific primer for V3–V4 region of 16S rRNA gene as described in the Illumina protocol, 0.75 U of Platinum Pfx DNA polymerase (C11708021, Invitrogen, United States), and 12 ng template DNA. The PCR cycle number was reduced to 21 to diminish bias. The program was started with pre-denaturation at 95°C for 3 min, followed by 22 cycles of denaturation at 94°C for 30 s, annealing at 55°C for 30 s and extension at 72°C for 30 s, and ended up with a final extension at 72°C for 5 min. For the Index PCR (attachment of dual indices and Illumina sequencing adapter using the Nextera XT Index Kit), the 25 μl reaction mix consisted of 1.0× Pfx amplification buffer, 1 mM MgSO_4_, 0.2 mM dNTP, 2.5 μl of each N7 and S5 Index primers as described in the protocol, 0.5 U of Platinum Pfx DNA polymerase, and 2.5 μl purified products of the Amplicon PCR step as template DNA. The PCR program of Index PCR was the same as Amplicon PCR except that the cycle number was reduced to 8. The purified products of the Index PCR were mixed at equal ratio and sequenced using the Illumina MiSeq System (Illumina Inc., United States).

### Bacterial Isolation and DNA Extraction

Bacteria were isolated from the breast milk inoculum and the feces of No. 4 recipient mouse at week 8. Wilkins-Chalgren Agar [WCH] (Hopebiol, Qingdao, China) as a non-selective medium for anaerobic bacteria (Jimenez et al., 2008; Jost et al., 2013) and M17 medium (Hopebiol, Qingdao, China) for streptococci, lactococci, and enterococci (Heikkila and Saris, 2003) were used for bacterial isolation. Three aliquots of 100 μl fresh milk or homogenized fecal suspension serially diluted by 10-fold were plated in triplicate on each agar medium. The plates were incubated in an anaerobic workstation (DG500, DWS, United Kingdom) at 37°C for 48 h. The bacterial population levels were reported as log colony-forming units (cfu)/ml breast milk or log cfu/g feces.

Based on different morphologies, colonies were randomly selected per sample and agar medium, streaked three times for purity and cultured in liquid Anaerobe Basal Broth medium [ABB] (Nissui, Qingdao, China). Aliquots of the suspension of viable isolates were stored at -80°C in liquid ABB medium supplemented with 30% (v/v) glycerol and covered by 300 μl paraffin oil.

To extract the genomic DNA from each viable bacterial isolate, 3 ml suspension of the isolate after 48 h cultivation was centrifuged at 9000 × *g* for 5 min. The bacterial cell pellets were re-suspended in 475 μl TE buffer (10 mM Tris-HCl, 1 mM EDTA, pH 8.0). Twenty-five microliter lysozyme (50 mg/ml) was added and the mixture was incubated at 37°C for 1 h. Then, 5 μl of Proteinase K (20 mg/ml) and 50 μl of 20% SDS were added and the mixture was incubated at 55°C for 30 min. The suspension was sequentially extracted by equal volumes of phenol, phenol/chloroform/isoamyl alcohol (vol/vol/vol 25:24:1), and chloroform/isoamyl alcohol (vol/vol, 24:1). DNA was precipitated with two volumes of ethanol at -20°C for 2 h, collected by centrifugation at 14,000 rpm for 15 min, washed twice with 500 μl ice-cold 70% (v/v) ethanol and air dried. DNA was re-suspended in 50 μl TE buffer. RNA was digested by adding 20 μl RNase (20 mg/ml) and incubating at 37°C for 30 min. The amount of DNA was determined with PicoGreen fluorescent dye (Thermo Fisher Scientific, Sunnyvale, CA, United States) by using SpectraMax M5 microplate reader (Molecular Devices, San Francisco, CA, United States), and its integrity was checked by 0.8% agarose gel electrophoresis stained by ethidium bromide.

### Enterobacterial Repetitive Intergenic Consensus Sequence-PCR

The bacterial isolates were genotyped by ERIC-PCR with the primer pair ERIC1 (5′-ATGTAAGCTCCTGGGGATTCAC-3′) and ERIC2 (5′-AAGTAAGTGACTGGGGTGAGCG-3′) (de Bruijn, 1992; Wei et al., 2004). The 25 μl PCR mixture contained 20 ng of bacterial genomic DNA, 200 mM each dNTP, 2.5 U of TaKaRa rTaq polymerase (Takara, Dalian, China), 1× reaction buffer, 2 mM MgCl_2_, and 10 pM of each primer. The amplification program was as follows: pre-denaturation at 95°C for 7 min, 30 cycles of denaturation at 95°C for 30 s, annealing at 52°C for 1 min, and extension at 65°C for 8 min, and a final extension at 65°C for 16 min. 400 ng of PCR products were separated by electrophoresis on a 1.5% (wt/vol) agarose gel.

### Full-Length 16S rRNA Genes Sequencing of Representative Bacterial Isolates

One representative isolate of each ERIC type was subjected to full-length 16S rRNA gene sequencing. Universal bacterial primers 27f (5′-AGAGTTTGATCCTGGCTCAG-3′) and 1492r (5′-CGGC/TTACCTTGTTACGACTT-3′) were used. The 25 μl mixture contained 0.75 U of rTaq polymerase (Takara, Dalian, China), 1× PCR buffer (Mg^2+^free), 2 mM MgCl_2_, 10 pmol of each primer, 200 μM each dNTP, and 10 ng of bacterial genomic DNA as the template. A 25 cycles PCR program was performed as follows: pre-denaturation at 95°C for 7 min, 30 cycles of denaturation at 94°C for 30 s, annealing at 52°C for 1 min, and extension at 65°C for 8 min, and a final extension at 65°C for 16 min.

PCR products were purified using the Gel Extraction Kit 200 (Omega, United States), ligated into the pGEM-T easy vector (Promega, Madison, WI, United States), and transformed into competent *E. coli* DH5a cells. Positive clones were picked randomly, amplified with T7 and SP6 as the primers. For each isolate, three positive clones were sequenced (Life Technologies, Shanghai, China).

### Bioinformatics Analysis

The 16S rRNA gene sequences obtained from the DGGE bands and bacterial isolates were blasted against the nr database of Genbank using the basic local alignment search tool (BLAST)^2^, and their closest relative bacteria were determined. A neighbor-joining phylogenetic tree containing the sequences and their relatives was constructed with the Molecular Evolutionary Genetics Analysis package (MEGA5) with the Jukes-Cantor algorithm. The phylogenetic robustness was assessed by bootstrap analysis with 1000 replicates using the same software. The taxonomy of the bacterial isolates and bacteria represented by the DGGE bands was determined based on the position of their sequences in the phylogenetic tree.

For the raw data of Illumina sequencing of 16S rRNA gene V3–V4 amplicons, both the forward and reverse ends of the same read were truncated at the first base where the Q value became no more than 2. Using USEARCH v8.0.1623, individual pairs of reads were merged into a complete read only if they had a minimum overlap of 50 bp. The merged reads that were longer than 399 nt with an expected error of no more than 0.5 were kept for further processing. Quality-filtered reads were delineated into unique sequences and then sorted by decreasing abundance, and singletons were discarded. OTUs were clustered *de novo* with Uparse (Edgar, 2013) at 97% similarity level. Reference-based chimera detection was performed using UCHIME (Edgar et al., 2011) against the RDP classifier training database (v9) (Cole et al., 2014). The OTU table was finalized by mapping quality-filtered reads to the remaining OTUs with the Usearch (Edgar, 2010) global alignment algorithm at a 97% cutoff. Sequence data were rarefied to 25,000 reads per sample (1,000 permutations) to avoid bias caused by the difference in sequencing depth. Representative sequences for each OTU were subjected to the RDP classifier to determine the taxonomy with a bootstrap cutoff of 80% (RDP database version 2.10).

### Accession Number

The full-length 16S rRNA gene sequences of the representative bacterial strains isolated from the breast milk and mouse feces and the 16S rRNA gene V3 region sequences of the DGGE band sequences were deposited in GenBank under accession numbers KY038179-KY038195 and KY082697-KY082707, respectively.

16S rRNA gene V3–V4 region Illumina sequences of the breast milk and mice feces were deposited in NCBI Sequence Read Archive (SRA) under accession numbers PRJNA351774 (breast milk), PRJNA351775 (mice at week 8), and PRJNA377923 (mice at weeks 1–6).

## Results

### The Colonization of Breast Milk Bacteria in Germ-Free Mice Monitored by DGGE

**FIGURE 1:**
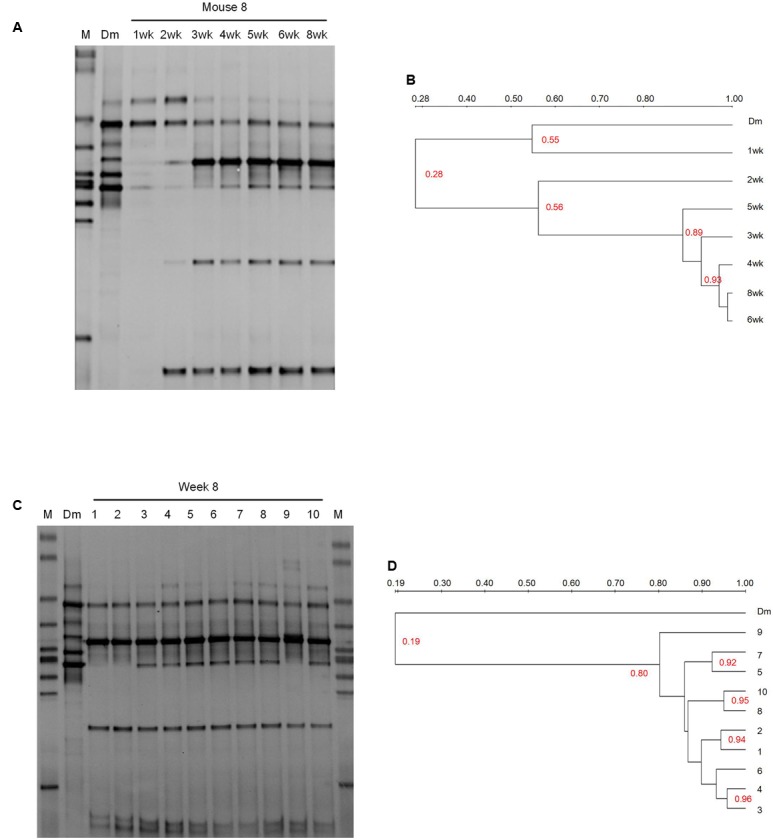
DGGE profiling of the bacterial communities in the breast milk inoculum and feces of recipient mice. **(A)** The weekly monitor of the gut microbiota composition of one recipient mouse (No. 8 mouse) after the inoculation of breast milk. **(B)** Dendrogram of the DGGE profiles shown in **(A)**. **(C)** The comparison of the bacterial composition between the breast milk inoculum and the feces of recipient mice at 8 weeks. **(D)** Dendrogram of the DGGE profiles shown in **(C)**. Dendrogram of the DGGE profiles was generated based on the similarity of profiles with UPGAMA clustering analysis using the Quantity One software. M, DGGE marker. Dm, breast milk. wk, week.

DGGE of 16S rRNA gene V3 region amplicons detects bacteria representing more than 1% of the whole community (Muyzer et al., 1993). We here performed DGGE and clustering analysis of the DGGE profiles to compare bacterial composition of the milk inoculum and the feces of recipient mice (**Figure 1** and Supplementary Figure S1).

The fecal microbiota of recipient mice became stabilized by 2–4 weeks after gavage according to clustering dendrogram of DGGE profiles of mice at different time points (**Figures 1A,B** and Supplementary Figure S1). At 8 weeks, the DGGE profiles of all 10 recipient mice clustered together with the similarity between 80 and 96%, but clustered separately from that of the breast milk with the similarity as low as 19% (**Figures 1C,D**). This suggests that despite the small inter-individual variation, the composition of gut microbiota of different recipient mice was similar but was significantly different from that of the breast milk inoculum.

**FIGURE 2:**
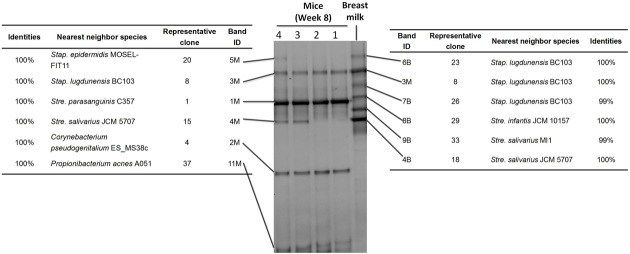
Sequence analysis of dominant bands in the 16S rRNA gene V3 region DGGE profiles of Mouse feces and Breast milk. The 16S rRNA gene V3 region DNA in each DGGE band was excised, re-amplified, cloned, and sequences. Indicated are the band ID, the representative clones, the bacterial species most closely related to the clones, and the levels of similarity.

The DGGE bands were excised, cloned, and sequenced (**Figure 2**). There were six bands in the profile of the breast milk; and among them, three were from *Staphylococcus lugdunensis*-like species, one from a *Streptococcus infantis*-like species, and two from *Streptococcus salivarius*-like species (**Figure 2**).

Only two breast milk bands (band 3M and 4M), from a *S. lugdunensis*-like species and a *Str. salivarius*-like species, respectively, were still present in the feces of the recipient mice. Four bands (5M, 1M, 2M, and 11M), representing *S. epidermidis*, *Str. parasanguinis*, *Corynebacterium pseudogenitalium*, and *Propionibacterium acnes*-like species, respectively, were not detectable in the breast milk, but appeared in mice (**Figure 2**). Band 4M and 5M (from a *Str. salivarius*-like and a *S. epidermidis*-like species, respectively) were detected in seven mice, and band 3M, 1M, 2M, and 11M (from a *S. lugdunensis*-like, *Str. parasanguinis*, *C. pseudogenitalium*, and *P. acnes*-like species, respectively) were detected in all 10 mice (**Figure 1**). The above six DGGE bands represented the breast milk bacteria that stably colonized the gut of recipient mice at the abundance > 1%.

### The Colonization of Breast Milk Bacteria in Germ-Free Mice Monitored with Illumina Sequencing of 16S rRNA Gene V3–V4 Region

The breast milk inoculum and feces of 10 recipient mice at weeks 1, 2, 3, 4, 5, 6, and 8 were subjected to Illumina sequencing of 16S rRNA gene V3–V4 regions, and 25366 and 46303 ± 26452 (median 34864, upper quartile 51414, and lower quartile 30531) high-quality reads were obtained for the milk and the mouse feces, respectively. The sequences were binned into OTUs at the 97% similarity level, and 165 OTUs were generated after chimeras and singleton filtering.

Thirty-seven OTUs were present in both the milk and Week 8 mouse feces (**Table 1** and Supplementary Table S1), 51 were detected only in the milk (**Table 2** and Supplementary Table S1), and 19 existed only in the mouse feces (**Table 3** and Supplementary Table S1).

**Table 1:** OTUs detected in both the breast milk inoculum and feces of recipient mice at week 8.

OTU ID	Phylum	Family	Genus	Abundance (%) in breast milk	Number of mice harboring the OTU§ (mouse ID)	Range of abundance (%) of the OTU in the mice harboring the OTU
OTU1	Firmicutes	Streptococcaceae	Streptococcus	7.524	10 (D1-10)	73.490–85.738
OTU4	Firmicutes	Streptococcaceae	Streptococcus	48.520	10 (D1-10)	0.027–6.294
OTU3	Actinobacteria	Corynebacteriaceae	Corynebacterium	0.004	10 (D1-10)	5.766–14.336
OTU2	Firmicutes	Staphylococcaceae	Staphylococcus	37.403	10 (D1-10)	5.639-11.144
OTU5	Actinobacteria	Propionibacteriaceae	Propionibacterium	0.020	10 (D1-10)	0.989–2.692
OTU6	Firmicutes	Ruminococcaceae	Faecalibacterium	0.754	4 (D3,4,5,10)	0.003–0.009
OTU11	Bacteroidetes	Prevotellaceae	Prevotella	0.028	3 (D2,6,10)	0.003–0.009
OTU8	Firmicutes	Lachnospiraceae	Roseburia	0.418	1 (D10)	0.006
OTU39	Firmicutes	Lachnospiraceae	Unclassified Lachnospiraceae	0.102	1 (D10)	0.009
OTU27	Firmicutes	Ruminococcaceae	Ruminococcus	0.008	1 (D10)	0.006
OTU13	Bacteroidetes	Bacteroidaceae	Bacteroides	0.015	1 (D10)	0.003
OTU37	Firmicutes	Lachnospiraceae	Blautia	0.070	2 (D4,10)	0.003
OTU29	Firmicutes	Erysipelotrichaceae	Clostridium XVIII	0.035	2 (D3,6)	0.003
OTU63	Bacteroidetes	Bacteroidaceae	Bacteroides	0.019	2 (D8,9)	0.003–0.009
OTU110	Firmicutes	Ruminococcaceae	Ruminococcus	0.004	1 (D8)	0.003
OTU28	Bacteroidetes	Bacteroidaceae	Bacteroides	0.019	1 (D3)	0.003
OTU9	Bacteroidetes	Bacteroidaceae	Bacteroides	0.039	2 (D9,10)	0.003
OTU12	Bacteroidetes	Bacteroidaceae	Bacteroides	0.012	1 (D10)	0.003
OTU117	Firmicutes	Lactobacillaceae	Lactobacillus	0.071	2 (D4,6)	0.003–0.007
OTU147	Firmicutes	Lactobacillaceae	Lactobacillus	0.016	2 (D7,10)	0.003–0.006
OTU146	Firmicutes	Lactobacillaceae	Lactobacillus	0.024	1 (D6)	0.003
OTU91	Bacteroidetes	Porphyromonadaceae	Parabacteroides	0.008	1 (D2)	0.003
OTU36	Firmicutes	Lachnospiraceae	Lachnospiracea_incertae_sedis	0.217	1 (D10)	0.003
OTU26	Firmicutes	Lachnospiraceae	Unclassified Lachnospiraceae	0.097	1 (D10)	0.003
OTU24	Firmicutes	Lachnospiraceae	Unclassified Lachnospiraceae	0.058	1 (D10)	0.003
OTU159	Firmicutes	Veillonellaceae	Veillonella	0.015	1 (D7)	0.003
OTU14	Proteobacteria	Idiomarinaceae	Aliidiomarina	0.799	4 (D1,6,8,9)	0.003
OTU15	Proteobacteria	Halomonadaceae	Halomonas	0.521	4 (D1,2,4,7)	0.003–0.006
OTU18	Actinobacteria	Dietziaceae	Dietzia	0.063	4 (D2,4,6,10)	0.003
OTU32	Actinobacteria	Nitriliruptoraceae	Nitriliruptor	0.016	4 (D2,6,8,9)	0.003–0.006
OTU16	Firmicutes	Lachnospiraceae	Fusicatenibacter	0.081	2 (D4,6)	0.003
OTU22	Actinobacteria	Nocardioidaceae	Aeromicrobium	0.039	2 (D5,9)	0.003
OTU88	Proteobacteria	Phyllobacteriaceae	Unclassified Phyllobacteriaceae	0.035	2 (D7,8)	0.003–0.009
OTU85	Proteobacteria	Xanthomonadaceae	Unclassified Xanthomonadaceae	0.031	2 (D2,8)	0.003
OTU23	Proteobacteria	Enterobacteriaceae	Unclassified Enterobacteriaceae	0.008	2 (D1,6)	0.003–0.009
OTU67	Firmicutes	Ruminococcaceae	Butyricicoccus	0.012	1 (D5)	0.003
OTU164	Cyanobacteria/Chloroplast	Chloroplast	Streptophyta	0.004	1 (D10)	0.003


*§Ten mice in total*.

**Table 2:** OTUs detected in the breast milk inoculum but not in the feces of recipient mice at week 8.

OTU ID	Phylum	Family	Genus	Abundance (%) in breast milk
OTU35	Proteobacteria	Comamonadaceae	Acidovorax	0.153
OTU43	Firmicutes	Ruminococcaceae	Ruminococcus	0.140
OTU20	Firmicutes	Ruminococcaceae	Gemmiger	0.140
OTU33	Firmicutes	Ruminococcaceae	Gemmiger	0.062
OTU25	Firmicutes	Veillonellaceae	Megamonas	0.113
OTU74	Firmicutes	Bacillales_Incertae Sedis XI	Gemella	0.113
OTU60	Proteobacteria	Burkholderiaceae	Ralstonia	0.074
OTU95	Proteobacteria	Pseudomonadaceae	Pseudomonas	0.062
OTU46	Firmicutes	Unclassified Clostridiales	Unclassified Clostridiales	0.062
OTU106	Firmicutes	Unclassified Clostridiales	Unclassified Clostridiales	0.039
OTU125	Firmicutes	Unclassified Clostridiales	Unclassified Clostridiales	0.012
OTU68	Proteobacteria	Burkholderiales_incertae_sedis	Aquabacterium	0.058
OTU40	Proteobacteria	Enterobacteriaceae	Escherichia/Shigella	0.058
OTU7	Firmicutes	Lachnospiraceae	Blautia	0.051
OTU157	Firmicutes	Lachnospiraceae	Blautia	0.008
OTU108	Proteobacteria	Oxalobacteraceae	Undibacterium	0.051
OTU92	Proteobacteria	Pasteurellaceae	Haemophilus	0.047
OTU128	Firmicutes	Lachnospiraceae	Unclassified Lachnospiraceae	0.046
OTU51	Firmicutes	Lachnospiraceae	Unclassified Lachnospiraceae	0.043
OTU76	Firmicutes	Lachnospiraceae	Unclassified Lachnospiraceae	0.027
OTU31	Firmicutes	Lachnospiraceae	Unclassified Lachnospiraceae	0.027
OTU62	Firmicutes	Lachnospiraceae	Unclassified Lachnospiraceae	0.008
OTU148	Firmicutes	Lachnospiraceae	Unclassified Lachnospiraceae	0.004
OTU21	Firmicutes	Acidaminococcaceae	Phascolarctobacterium	0.043
OTU113	Firmicutes	Veillonellaceae	Dialister	0.043
OTU133	Firmicutes	Veillonellaceae	Veillonella	0.031
OTU104	Actinobacteria	Micrococcaceae	Nesterenkonia	0.031
OTU70	Firmicutes	Ruminococcaceae	Butyricicoccus	0.031
OTU42	Firmicutes	Lachnospiraceae	Anaerostipes	0.027
OTU118	Actinobacteria	Micrococcaceae	Rothia	0.023
OTU10	Fusobacteria	Fusobacteriaceae	Fusobacterium	0.023
OTU49	Firmicutes	Lachnospiraceae	Dorea	0.023
OTU103	Firmicutes	Lachnospiraceae	Lachnospiracea_incertae_sedis	0.020
OTU48	Firmicutes	Lachnospiraceae	Lachnospiracea_incertae_sedis	0.015
OTU47	Firmicutes	Lachnospiraceae	Lachnospiracea_incertae_sedis	0.004
OTU89	Proteobacteria	Pasteurellaceae	Haemophilus	0.020
OTU61	Firmicutes	Unclassified Firmicutes	Unclassified Firmicutes	0.016
OTU44	Firmicutes	Veillonellaceae	Megasphaera	0.016
OTU79	Proteobacteria	Sutterellaceae	Parasutterella	0.011
OTU65	Bacteroidetes	Prevotellaceae	Paraprevotella	0.008
OTU123	Bacteroidetes	Prevotellaceae	Paraprevotella	0.008
OTU126	Firmicutes	Erysipelotrichaceae	Clostridium XVIII	0.008
OTU151	Firmicutes	Clostridiaceae 1	Clostridium sensu stricto	0.008
OTU72	Bacteroidetes	Rikenellaceae	Alistipes	0.008
OTU119	Firmicutes	Lachnospiraceae	Clostridium XlVb	0.007
OTU134	Firmicutes	Streptococcaceae	Streptococcus	0.004
OTU71	Firmicutes	Ruminococcaceae	Unclassified Ruminococcaceae	0.004
OTU82	Firmicutes	Ruminococcaceae	Flavonifractor	0.004
OTU153	Firmicutes	Lachnospiraceae	Coprococcus	0.004
OTU115	Firmicutes	Ruminococcaceae	Oscillibacter	0.004
OTU53	Firmicutes	Peptostreptococcaceae	Romboutsia	0.004


**Table 3:** OTUs detected only in the feces of recipient mice at week 8 but not in the breast milk inoculum.

OTU ID	Phylum	Family	Genus	Abundance (%) in breast milk	Number of mice harboring the OTUaaa	Range of abundance (%) of the OTU in the mice harboring the OTU
OTU94	Firmicutes	Streptococcaceae	Streptococcus	0	5	0.003–0.006
OTU55	Actinobacteria	Bifidobacteriaceae	Bifidobacterium	0	3	0.003–0.006
OTU19	Actinobacteria	Bifidobacteriaceae	Bifidobacterium	0	2	0.003
OTU130	Proteobacteria	Rhodocyclaceae	Dechloromonas	0	2	0.003–0.006
OTU83	Actinobacteria	Bogoriellaceae	Bogoriella	0	2	0.003
OTU57	Verrucomicrobia	Verrucomicrobiaceae	Akkermansia	0	1	0.006
OTU137	Proteobacteria	Alcanivoracaceae	Alcanivorax	0	1	0.003
OTU52	Bacteroidetes	Rikenellaceae	Alistipes	0	1	0.003
OTU38	Bacteroidetes	Bacteroidaceae	Bacteroides	0	1	0.003
OTU75	Bacteroidetes	Bacteroidaceae	Bacteroides	0	1	0.003
OTU121	Actinobacteria	Coriobacteriaceae	Collinsella	0	1	0.003
OTU45	Firmicutes	Lachnospiraceae	Coprococcus	0	1	0.003
OTU86	Firmicutes	Erysipelotrichaceae	Holdemanella	0	1	0.003
OTU112	Bacteroidetes	Porphyromonadaceae	Odoribacter	0	1	0.003
OTU150	Bacteroidetes	Porphyromonadaceae	Parabacteroides	0	1	0.003
OTU34	Bacteroidetes	Prevotellaceae	Prevotella	0	1	0.003
OTU154	Proteobacteria	Rhodocyclaceae	Thauera	0	1	0.003
OTU59	Actinobacteria	Microbacteriaceae	Unclassified Microbacteriaceae	0	1	0.003
OTU129	Firmicutes	Ruminococcaceae	Unclassified Ruminococcaceae	0	1	0.003


*§Ten mice in total*.

Three OTUs from *Staphylococcus* (OTU2) and *Streptococcus* (OTU1 and OTU4), were among the most abundant bacteria in both the milk and feces of 10 mice at week 8; the abundance of *Staphylococcus* decreased from 37.4% in the milk to 7.6 ± 1.6% (range 5.6–11.1%) in the feces of recipient mice, in contrast, the abundance of *Streptococcus* (OTU1 and OTU4) increased from 56.0% in the milk to 80.3 ± 2.3% (range 77.6–86.3%) in the mouse feces (**Table 1**). Two OTUs, from *Corynebacterium* (OTU3) and *Propionibacterium* (OTU5), respectively, accounted for only 0.004 and 0.02% in the breast milk, but became much more abundant in 10 mice at week 8 with the abundance 10.0 ± 2.6% (range 5.8–14.3%) and 2.1 ± 0.5% (range 1.0–2.7%), respectively (**Table 1**). In the gut of recipient mice at week 8, only the above five OTUs were at abundance > 1%, and the abundance of *Streptococcus* (range 77.6–86.3%), *Corynebacterium* (range 5.8–14.3%), *Staphylococcus* (range 5.6–11.1%), and *Propionibacterium* (range 1–2.7%) generally decreased sequentially (**Table 1**). The above 5 OTUs were detected in all 10 mice at most of the time points (>5) from weeks 1 to 8, and their abundances remained stable from weeks 6 to 8 (Supplementary Figure S2 and Table S2).

Some gut-associated anaerobic bacteria from *Faecalibacterium* (OTU6), *Prevotella* (OTU11), *Roseburia* (OTU8), Unclassified Lachnospiraceae (OTU39), *Ruminococcus* (OTU27), and *Bacteroides* (OTU13) were present at low abundance in the milk inoculum (0.01–1%) and in the feces of some but not all recipient mice at week 8 (0.003–0.01%) (**Table 1**). These bacteria were detected at multiple time points from weeks 1 to 8 in mice harboring them. *Faecalibacterium* (OTU6) was detected at 3–5 time points in four mice, *Prevotella* (OTU11) was detected at 4–5 time points in three mice, and *Roseburia* (OTU8), Unclassified Lachnospiraceae (OTU39), *Ruminococcus* (OTU27), and *Bacteroides* (OTU13) were present in one mouse at 2–3 time points (Supplementary Figure S3 and Table S2). Moreover, their abundances stayed at 0.003–0.01% from weeks 6 to 8 (Supplementary Figure S3 and Table S2).

Some bacteria, such as *Gemella* (OTU74), *Dialister* (OTU113), *Dorea* (OTU49), were detected only in the breast milk at abundance 0.01–0.1%, but not in any recipient mice (**Table 2**).

Bacteria from *Bifidobacterium*, represented by OTU55 and OTU19, were not detected in the breast milk inoculum, but were detected at abundance 0.003–0.01% in some recipient mice at week 8 (**Table 3**). In one mouse (D7), the two *Bifidobacterium* OTUs were repeatedly detected from weeks 4 to 8 (Supplementary Figure S3 and Table S2).

### Isolation of Bacteria from the Breast Milk Inoculum and the Feces of One Recipient Mouse

Bacteria were isolated from the breast milk inoculum and the feces of No. 4 recipient mouse at week 8. The No.4 mouse was selected because its DGGE profile at week 8 contained all the six dominant bands detected in other recipient mice (**Figure 1**). WCH and M17 media were used because previous studies showed the majority of bacteria isolated from human breast milk grew on WCH medium (Jost et al., 2013), and streptococci, lactococci, and enterococci grew better on M17 than on MRS agar plates (Heikkila and Saris, 2003).

The viable bacterial counts in the breast milk inoculum were log 5.18 ± 0.15 cfu/ml (range log 4.97–5.3 cfu/ml) and log 3.97 ± 0.09 cfu/ml (range log 3.9–4.1 cfu/ml) as detected by WCH and M17, respectively. The bacterial counts of mouse feces were 9.28 ± 0.23 cfu/g (range log 8.87–9.54 cfu/g) and log 9.49 ± 0.28 cfu/g (range log 9.11–9.78 cfu/g) as detected by WCH and M17, respectively.

One hundred and eighty-six isolates (148 on WCH, and 40 on M17) and 282 isolates (119 on WCH, and 163 on M17) were isolated from the breast milk inoculum and the mouse feces, respectively. With ERIC PCR, the 186 isolates of the breast milk were genotyped into 12 different ERIC types (BM-E1 to BM-E12), and the 282 isolates of the mouse feces were classified into 5 ERIC types (MF-E13 to MF-E17) (Supplementary Figure S4). The breast milk and mouse feces isolates shared no common ERIC type (Supplementary Figure S4).

**FIGURE 3:**
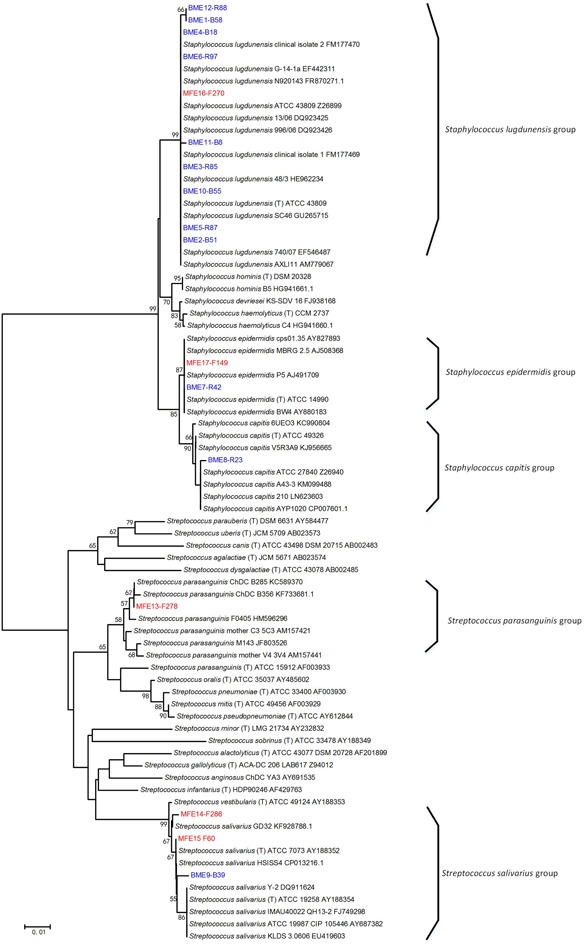
Phylogenetic tree of the representative bacterial isolates of 17 ERIC types and other known bacteria. The tree was constructed based on the region from base 27 to base 1492 of the 16S rRNA genes. The ERIC types isolated from breast milk (BME 1–12) and from the feces of No. 4 recipient mouse (MFE 13–17) are indicated by blue and red font, respectively, and the ID of the representative strain of each ERIC type was written after. Bacterial strains retrieved from the GenBank database are indicated by italics, and their accession numbers are given. Bootstrap values greater than 50% are indicated at the nodes.

The taxonomy of the isolates was determined at the species-level by constructing a phylogenetic tree with the full-length 16S rRNA gene sequences of the representative strains of individual ERIC types (**Figure 3**), and the abundance of each bacterial species in the original sample was calculated as its percentage accounting for the total number of all isolates of the sample. Among the breast milk isolates, 90.9% were *Staphylococcus* species and only 9.1% were *Streptococcus*. Among the mouse fecal isolates, however, the percentage of S*taphylococcus* spp. was greatly reduced to 15.2%, and *Streptococcus* isolates accounted for as much as 84.7% (**Table 4**). This is consistent with the 16S rRNA gene Illumina sequencing result.

**Table 4:** The taxonomy and abundance of the bacteria isolated from the breast milk inoculum and the feces of No. 4 mouse at week 8.

Genus	Speciesaaa	Abundance in breast milk (%)ddddccccbbbb	Abundance in mouse feces (%)ddddccccbbbb
*Staphylococcus*		90.9	15.2
	*S. lugdunensis*	79	13.8
	*S. epidermidis*	9.7	1.4
	*S. capitis*	2.2	/
*Streptococcus*		9.1	84.7
	*Str. salivarius*	9.1	19.1
	*Str. parasanguinis*	/	65.6


*§The taxonomy of the bacteria is determined based on the phylogenetic tree constructed with the full-length 16S rRNA gene of the isolated strains and those deposited in GenBank (**Figure 3**). ddddccccbbbbThe abundance of individual bacterial species is the percentage of the species accounting for the total isolated strains from the sample these bacteria were isolated. One hundred and eighty-six and 282 strains were isolated from the breast milk inoculum and mouse feces, respectively*.

In accordance with the DGGE result, bacterial isolates belonging to *Str. parasanguinis* were not obtained from the milk, but were the most abundant isolates in the mouse feces (**Table 4**). The sequence of 16S rRNA gene V3–V4 region of the representative *Str. parasanguinis* isolate was compared to the representative sequences of OTUs belonging to *Streptococcus* from the Illumina sequencing results of 16S rRNA gene of the milk and mouse feces. It showed 99% similarity to the representative sequence of OTU1, but only 97% similarity to OTU4 and OTU134. These results suggest *Str. parasanguinis* was classified within OTU1 from the Illumina sequencing results of 16S rRNA gene of the milk and mouse feces.

## Discussion

Our results suggest that typically gut-associated bacteria detectable in human breast milk are alive and could stably colonize the intestine of germ-free mice. The breast milk bacteria that became most abundant (>1%) in all 10 recipient mice were *Streptococcus* (80.3 ± 2.3%), *Corynebacterium* (10 ± 2.6%), *Staphylococcus* (7.6 ± 1.6%), and *Propionibacterium* (2.1 ± 0.5%). These bacteria are among the first colonizers in the colon of human infants within the first weeks of life (Palmer et al., 2007; Backhed et al., 2015), and they have been shown to dominate the initial gut microbiota of C-section-delivered human babies (Dominguez-Bello et al., 2010), despite that their predominance in the gut persists only in the first weeks of life (Palmer et al., 2007; Backhed et al., 2015; Charbonneau et al., 2016). Of note, *Streptococcus* spp. are also prevalent species in the gut of Chinese children and adults, and the abundance can reach as high as 5–9% in some Chinese individuals according to Illumina sequencing of fecal 16S rRNA gene fragments (Zhang C. et al., 2015; Zhang J. et al., 2015). Bacteria within *Faecalibacterium*, *Prevotella*, *Roseburia*, Unclassified Lachnospiraceae, *Ruminococcus*, and *Bacteroides* are dominant gut bacteria in children and adults (Eckburg et al., 2005; Arumugam et al., 2011), and they were present in the breast milk inoculum and were repeatedly detected at multiple time points in the feces of some recipient mice. *Bifidobacterium* spp. have been identified as important bacteria that are vertically transferred from maternal breast milk to infant gut in humans (Martin et al., 2012; Jost et al., 2014). In the present study, the breast milk inoculum was the only source of commensal bacteria for the recipient mice, and bifidobacteria were detected in the feces of recipient mice but not in the breast milk inoculum with Illumina sequencing of 16S rRNA gene. This suggests that the gut of our breast milk microbiota-associated mice enriched bifidobacteria despite their abundance in the milk inoculum below the detection limit of Illumina sequencing of 16S rRNA gene. Among the bacteria discussed above, except *Staphylococcus* and *Bifidobacterium* (Martin et al., 2012; Jost et al., 2014), were those previously identified to be present in human breast milk, but direct evidence for their colonization of the infant gut has been lacking. Therefore, our results indicate the breast milk microbiota-associated mouse model can be used to identify additional breast milk bacteria that have the potential to colonize infant gut.

In human neonates, *Propionibacterium* spp. were found to be one of the pioneer colonizers in the gut (Dominguez-Bello et al., 2010; Backhed et al., 2015), but could not be detected in their mothers’ gut (Backhed et al., 2015). In the present study, the human breast milk *Propionibacterium* spp. were able to colonize the gut of germ-free mice, suggesting that breast milk may be one of sources of infant gut *Propionibacterium*.

*Streptococcus* spp. and *Staphylococcus* spp. are facultative anaerobes, and *Corynebacterium* and *Propionibacterium* genera include both aerobic and facultatively anaerobic species (Pascual et al., 1995; Collins and Cummins, 2005; Stackebrandt et al., 2006), and they were the only four genera at >1% abundance in the gut of our recipient mice. The very low abundance of *Corynebacterium* and *Propionibacterium* in the milk inoculum, which was as low as 0.004 and 0.02%, respectively, did not hamper them blooming in the gut of recipient mice. In contrast, the obligatory anaerobes, *Faecalibacterium*, *Prevotella*, *Roseburia*, Unclassified Lachnospiraceae, *Ruminococcus*, and *Bacteroides* stayed at very low abundance in recipient mice (0.003–0.01%). The dominance of aerobic or facultative anaerobic bacteria over obligatory anaerobes in the colon of our recipient mice resemble the observation of gut microbiota composition of human neonates in the first week of life (Palmer et al., 2007), and this is probably because the intestine of both germ-free mice (Celesk et al., 1976) and human newborns younger than 1-week-old is still in an aerobic condition. However, while the aerobic or facultative anaerobic bacteria are replaced by obligatory anaerobic bacteria within first weeks of life in the gut microbiota of human infants (Palmer et al., 2007), the fecal microbiota of our recipient mice stabilized with the dominance of aerobic or facultative anaerobic bacteria for 4–6 weeks in different mice. This indicates that factors that promote the growth of obligatory anaerobes in the gut are lacking for the breast milk microbiota-associated mice.

Diet exerts a determinant effect in shaping the composition of gut microbiota. In human infants, breast feeding results in bifidobacteria-dominating gut microbiota (Charbonneau et al., 2016), whereas formula feeding makes the infant gut microbiota an “adult-like microbiota” in which *Bacteroides*, members of the *Clostridium coccoides* group, and *Lactobacillus* are all predominantly represented (Fallani et al., 2010). In human adults, prebiotic inulin ingestion significantly increases the abundance of *Faecalibacterium prausnitzii* (Ramirez-Farias et al., 2009). In future studies, it would be worthwhile to feed the breast milk microbiota-associated mice with formula and prebiotics, which may enrich obligatory anaerobes in the gut of these mice.

This breast milk microbiota-associated mouse model might provide an alternative way to isolate the gut-associated bacteria from the breast milk. In previous studies using varying culture media, about 60% of the bacterial isolates of the breast milk were *Staphylococcus* spp. (Heikkila and Saris, 2003; Jimenez et al., 2008; Jost et al., 2013, 2014), which are prevalent and dominant on human skin (Grice et al., 2009). In agreement with these previous findings that bacterial isolates of the breast milk were predominantly *Staphylococcus*, the bacterial cultivation in the present study showed *Staphylococcus* accounted for 90.9% of the isolates of the breast milk. In contrast, in the feces of our breast milk microbiota-associate mice, the percentage of *Staphylococcus* isolates decreased to only 15.2%, and isolates of *Streptococcus* bacteria, which are prevalent in the gut microbiota of Chinese people (Zhang C. et al., 2015; Zhang J. et al., 2015), accounted for 84.7%. Furthermore, our results showed that the facultative anaerobic gut-associated bacteria from the breast milk inoculum stably colonized the gut of recipient mice, and that obligatory anaerobes were repeatedly detected at multiple time points in some recipient mice. These results suggest breast milk microbiota-associated mice can serve as sustainable carriers for these bacteria and can continuously provide feces for isolation of these bacteria.

In conclusion, our results showed the typically gut-associated bacteria in human breast milk could colonize the gut of germfree mice, and this breast milk microbiota-associated mouse model may be used to identify additional breast milk bacteria that can colonize the gut and are thus potentially involved in human mother-infant bacterial transfer via breast feeding.

## Author Contributions

LZ and JS designed the study; XW, HL, XX, JC, and CF collected the breast milk sample; XW performed the experiments; XW, ZF, and JS analyzed the data; XW, LZ, and JS wrote and revised the manuscript.

## Footnotes

1 http://support.illumina.com.cn/content/dam/illumina-support/documents/documentation/chemistry_documentation/16s/16s-metagenomic-library-prep-guide-15044223-b.pdf">

2 https://blast.ncbi.nlm.nih.gov/Blast.cgi?PROGRAM=blastn&PAGE_TYPE=BlastSearch&LINK_LOC=blasthome
