# Supplementary material for: Development of Human Breast Milk Microbiota-Associated Mice as a Method to Identify Breast Milk Bacteria Capable of Colonizing Gut
Source: Front Microbiol. 2017 Jul 11;8:1242. doi: 10.3389/fmicb.2017.01242 (PMC5504100; doi:10.3389/fmicb.2017.01242)
Supplement: Supplementary file 1 [file Data_Sheet_1.docx]

**Development of human breast milk microbiota-associated mice as a method to identify breast milk bacteria capable of colonizing gut**

Xiaoxin Wang, Huifang Lu, Zhou Feng, Jie Cao,Chao Fang, Xianming Xu, Liping Zhao, Jian Shen

**Supplementary Information**

**Summary**

The supplementary information includes supplementary materials and methods, four supplementary figures and two supplementary tables.

**Supplementary Materials and Methods**

**DNA extraction from breast milk and mouse feces**

The cell pellets from 2 ml breast milk and from one fecal pellet of recipient mice were re-suspended in 250 μl of 4 M guanidine thiocyanate–0.1 M Tris (pH 7.5) and 40 μl of 10% N-lauroyl sarcosine and 500 μl of 5% N-lauroyl sarcosine–0.1 M phosphate buffer (pH 8.0). All the material was transferred to a 2 ml screw-cap polypropylene microcentrifuge tube and was incubated at 70°C for 1 h. One volume (750 μl) of 0.1-mm-diameter silica beads previously sterilized by autoclaving was added, and the tube was shaken at 25hz/s for 10 min in a Vibro shaker (Retsch) tissue lyser. Polyvinylpolypyrroli-done (15 mg) was added to the tube, which was vortexed and centrifuged for 5 min at 14,000 rpm at 4℃. After recovery of the supernatant, the pellet was washed with 500ul of TENP (50 mM Tris [pH 8], 20 mM EDTA [pH 8], 100 mM NaCl, 1% polyvinylpolypyrrolidone) and centrifuged for 5 min at 14,000 rpm, and the new supernatant was added to the first supernatant. The washing step was repeated three times. The pooled supernatants (about 2 ml) were briefly centrifuged to remove particles and then split into two 2-ml tubes.

Nucleic acids were precipitated by the addition of 1 volume of isopropanol for 10 min at room temperature and centrifuged for 10 min at 14000rpm. Pellets were re-suspended and pooled in 450ul of 100 mM phosphate buffer, pH 8, and 50ul of 5 M potassium acetate to precipitate proteins mixed with the nucleic acids. The tube was placed on ice for 90 min and centrifuged at 14,000rpm for 30 min. The supernatant was transferred to a new 1.5-mL Tube containing 3uL of RNase (10 mg/ml) and incubated at 37°C for 30 min. Nucleic acids were precipitated by the addition of 50ul of 3 M sodium acetate and 1 ml of absolute ethanol. The tube was incubated for 2h at -20℃, and nucleic acids were recovered by centrifugation at 14,000rpm for 10 min. The DNA pellet was finally washed with 70% ethanol(stored at -20℃), dried, and re-suspended in 100ul of TE buffer.

**Supplementary Figures**

**Figure S1.**


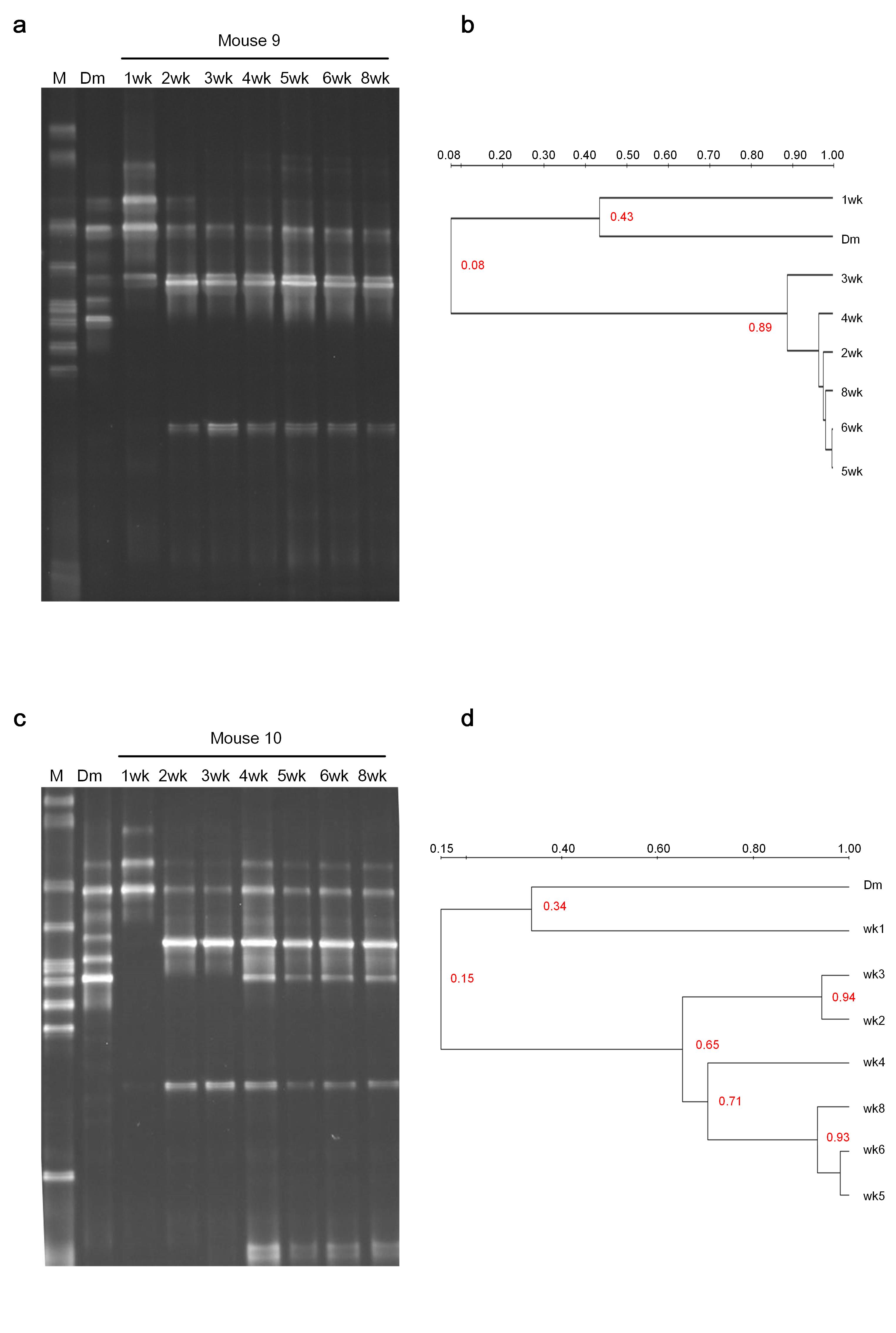


**Figure S1.** DGGE profiling of the bacterial communities in the breast milk inoculum and feces of recipient mice. (a)(c) The weekly monitor of the gut microbiota composition of two recipient mice (No. 9 mouse and No. 10 mouse respectively) after the inoculation of breast milk. (b)(d) Dendrogram of the DGGE profiles shown in (a) (c) respectively. Dendrogram of the DGGE profiles was generated based on the similarity of profiles with UPGAMA clustering analysis using the Quantity One software. M, DGGE marker. Dm, breast milk. wk, week.

**Figure S2.**

**
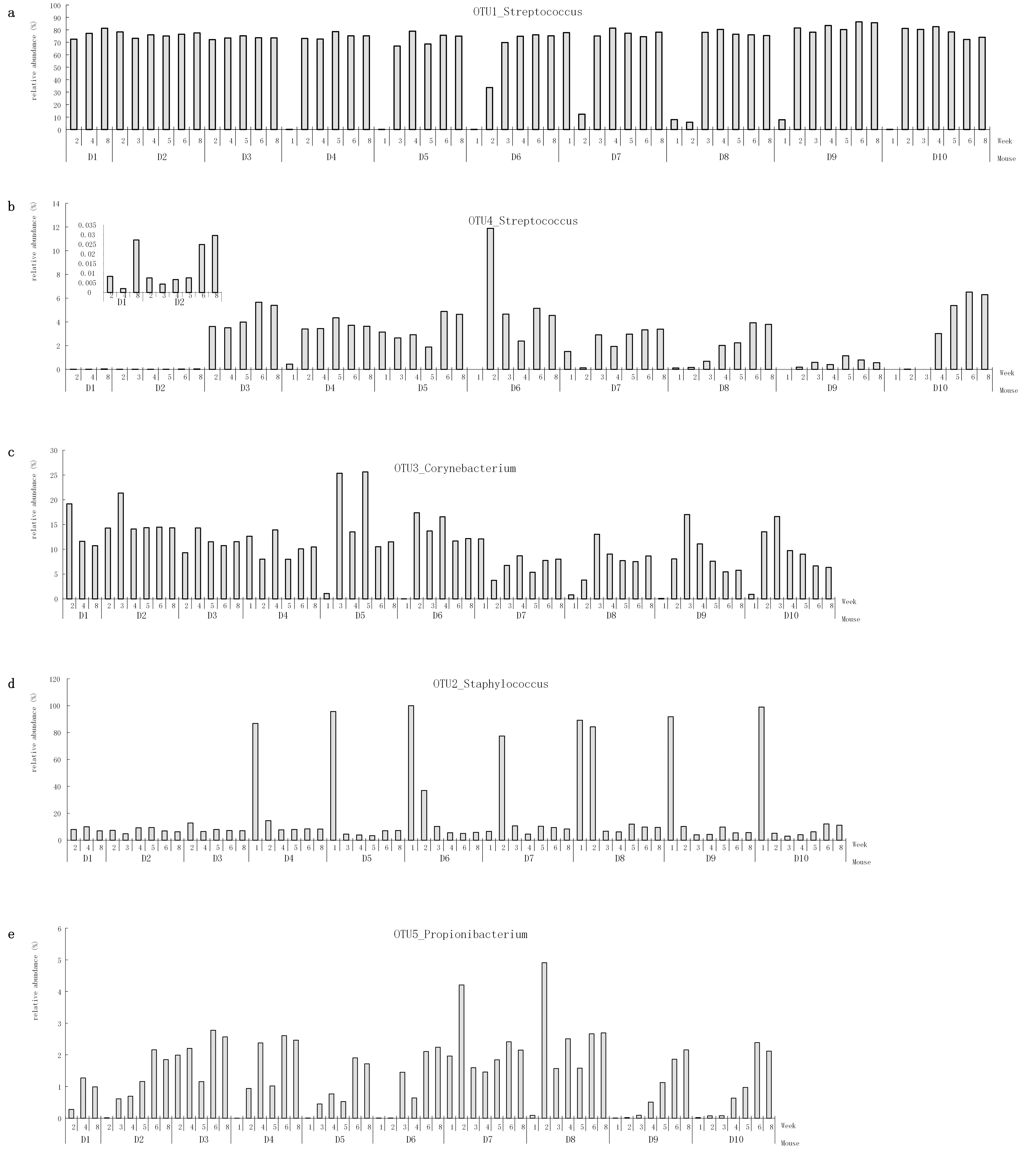
**

**Figure S2.** The abundance of OTU at different time points (week) that were detected at week 8 with the abundance >1% in the mice. The results were generated based on Illumina sequencing of 16S rRNA gene V3–V4 regions. (a) OTU1_Streptococcus. (b) OTU4_Streptococcus. The insert shows an enlarged view of the bars for mouse D1 and mouse D2. (c) OTU3_Corynebacterium. (d) OTU2_Staphylococcus. (e) OTU5_Propionibacterium.

**Figure S3.**

**
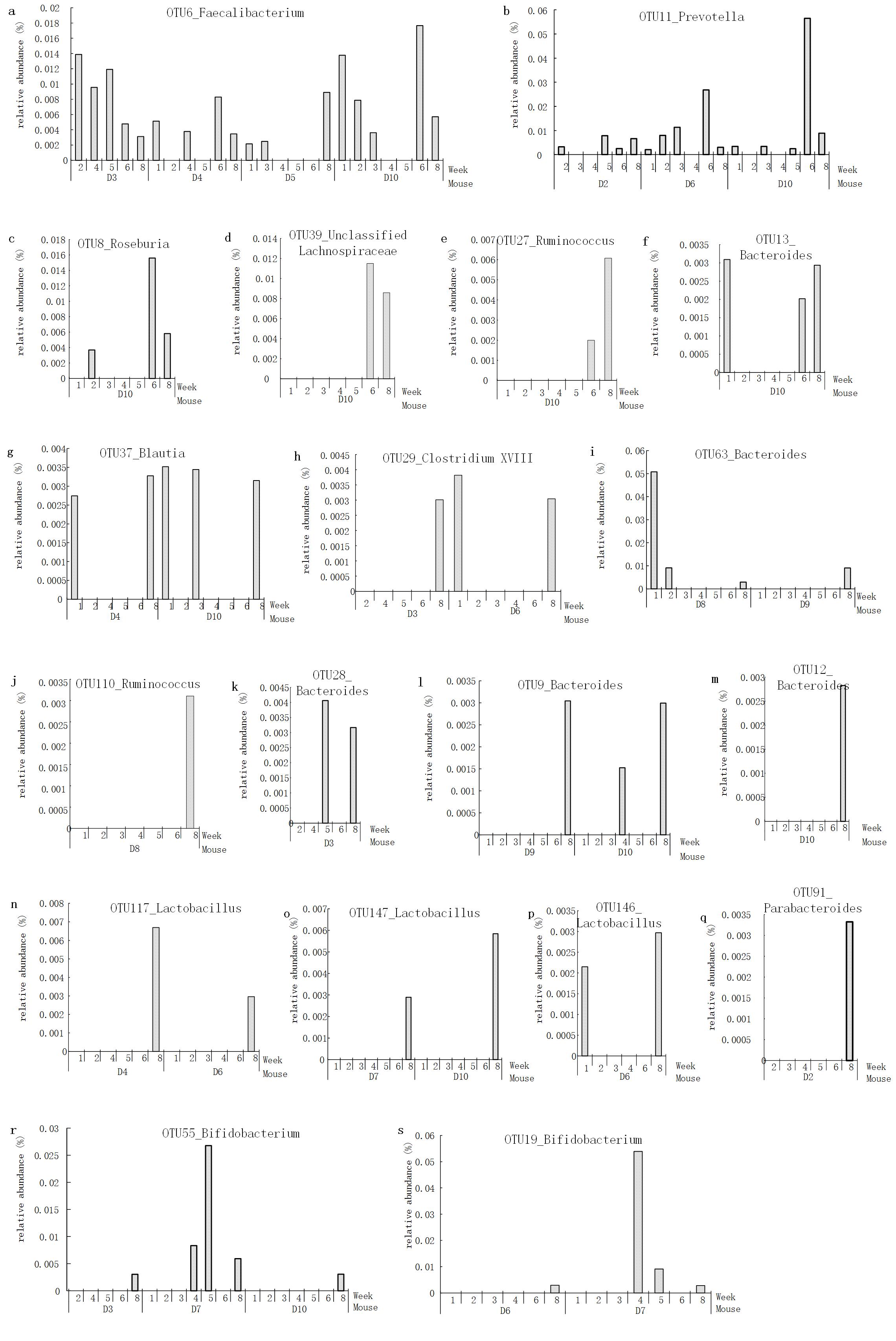
**

**Figure S3.** The abundance of OTUs at different time points (week) that were detected at week 8 with the abundance 0.003-0.01% in the mice (a-s). The results were generated based on Illumina sequencing of 16S rRNA gene V3–V4 regions.

**Figure S4.**


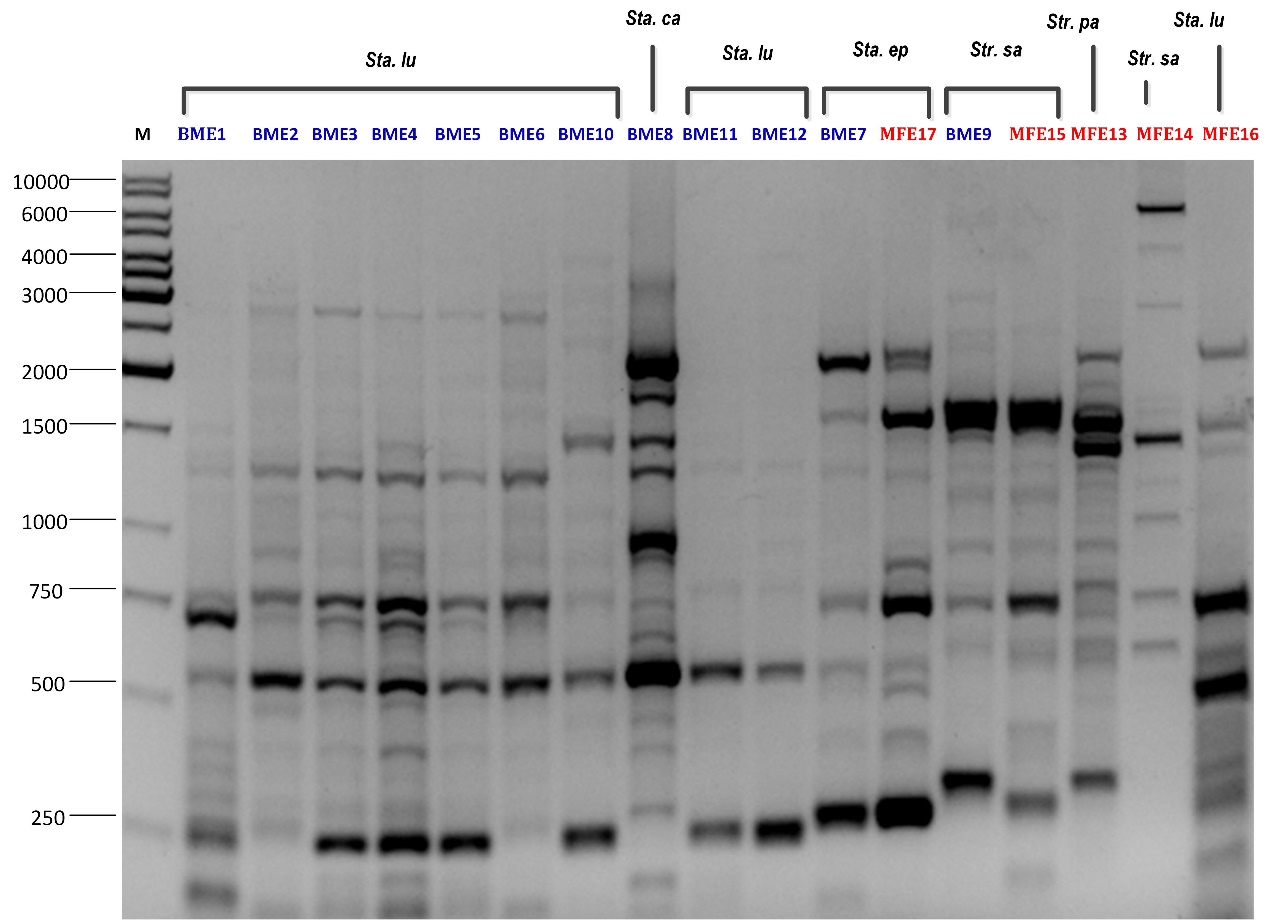


**Figure S4.** The ERIC-PCR fingerprinting types of bacteria isolated from the human breast milk inoculum and the feces of No. 4 recipient mouse. The ERIC types of bacteria isolated from the breast milk (BM-E1 to BM-E12) and from the mouse feces (MF-E13 to MF-E17) were labeled in blue and red, respectively, and above is the taxonomy of the bacteria of each ERIC type as determined by full-length 16S rRNA gene. *Sta. lu, Staphylococcus lugdunensis; Sta. ca, Staphylococcus capitis; Sta. ep, Staphylococcus epidermidis; Str. sa, Streptococcus salivarius; Str. pa, Streptococcus parasanguinis.* Lane M is the DNA marker, and the sizes (bp) of marker bands were indicated at the left.

**Supplementary Table S1.** The abundance of OTUs detected in the breast milk inoculum and feces of ten recipient mice at week 8

| **#OTU ID** | **Taxonomical assignments (RDP Classifier)** | | |  | |  | |  | |  | |  | |  | |  | |  | |  | |  | |  |
| --- | --- | --- | --- | --- | --- | --- | --- | --- | --- | --- | --- | --- | --- | --- | --- | --- | --- | --- | --- | --- | --- | --- | --- | --- |
|  | **Phylum** | **Family** | **Genus** | **Dm** | **D1** | | **D2** | | **D3** | | **D4** | | **D5** | | **D6** | | **D7** | | **D8** | | **D9** | | **D10** | |
| OTU1 | Firmicutes | Streptococcaceae | Streptococcus | 7.524 | 81.290 | | 77.542 | | 73.490 | | 75.166 | | 74.952 | | 75.225 | | 78.087 | | 75.374 | | 85.738 | | 73.988 | |
| OTU4 | Firmicutes | Streptococcaceae | Streptococcus | 48.520 | 0.027 | | 0.030 | | 5.391 | | 3.631 | | 4.633 | | 4.548 | | 3.381 | | 3.786 | | 0.559 | | 6.294 | |
| OTU3 | Actinobacteria | Corynebacteriaceae | Corynebacterium | 0.004 | 10.725 | | 14.336 | | 11.531 | | 10.479 | | 11.501 | | 12.173 | | 8.015 | | 8.651 | | 5.766 | | 6.357 | |
| OTU2 | Firmicutes | Staphylococcaceae | Staphylococcus | 37.403 | 6.951 | | 6.213 | | 6.994 | | 8.229 | | 7.171 | | 5.772 | | 8.330 | | 9.471 | | 5.639 | | 11.144 | |
| OTU5 | Actinobacteria | Propionibacteriaceae | Propionibacterium | 0.020 | 0.989 | | 1.850 | | 2.566 | | 2.462 | | 1.716 | | 2.240 | | 2.151 | | 2.692 | | 2.157 | | 2.117 | |
| OTU6 | Firmicutes | Ruminococcaceae | Faecalibacterium | 0.754 | 0.000 | | 0.000 | | 0.003 | | 0.003 | | 0.009 | | 0.000 | | 0.000 | | 0.000 | | 0.000 | | 0.006 | |
| OTU11 | Bacteroidetes | Prevotellaceae | Prevotella | 0.028 | 0.000 | | 0.007 | | 0.000 | | 0.000 | | 0.000 | | 0.003 | | 0.000 | | 0.000 | | 0.000 | | 0.009 | |
| OTU8 | Firmicutes | Lachnospiraceae | Roseburia | 0.418 | 0.000 | | 0.000 | | 0.000 | | 0.000 | | 0.000 | | 0.000 | | 0.000 | | 0.000 | | 0.000 | | 0.006 | |
| OTU39 | Firmicutes | Lachnospiraceae | Unclassified | 0.102 | 0.000 | | 0.000 | | 0.000 | | 0.000 | | 0.000 | | 0.000 | | 0.000 | | 0.000 | | 0.000 | | 0.009 | |
| OTU27 | Firmicutes | Ruminococcaceae | Ruminococcus | 0.008 | 0.000 | | 0.000 | | 0.000 | | 0.000 | | 0.000 | | 0.000 | | 0.000 | | 0.000 | | 0.000 | | 0.006 | |
| OTU13 | Bacteroidetes | Bacteroidaceae | Bacteroides | 0.015 | 0.000 | | 0.000 | | 0.000 | | 0.000 | | 0.000 | | 0.000 | | 0.000 | | 0.000 | | 0.000 | | 0.003 | |
| OTU37 | Firmicutes | Lachnospiraceae | Blautia | 0.070 | 0.000 | | 0.000 | | 0.000 | | 0.003 | | 0.000 | | 0.000 | | 0.000 | | 0.000 | | 0.000 | | 0.003 | |
| OTU29 | Firmicutes | Erysipelotrichaceae | Clostridium XVIII | 0.035 | 0.000 | | 0.000 | | 0.003 | | 0.000 | | 0.000 | | 0.003 | | 0.000 | | 0.000 | | 0.000 | | 0.000 | |
| OTU63 | Bacteroidetes | Bacteroidaceae | Bacteroides | 0.019 | 0.000 | | 0.000 | | 0.000 | | 0.000 | | 0.000 | | 0.000 | | 0.000 | | 0.003 | | 0.009 | | 0.000 | |
| OTU110 | Firmicutes | Ruminococcaceae | Ruminococcus | 0.004 | 0.000 | | 0.000 | | 0.000 | | 0.000 | | 0.000 | | 0.000 | | 0.000 | | 0.003 | | 0.000 | | 0.000 | |
| OTU28 | Bacteroidetes | Bacteroidaceae | Bacteroides | 0.019 | 0.000 | | 0.000 | | 0.003 | | 0.000 | | 0.000 | | 0.000 | | 0.000 | | 0.000 | | 0.000 | | 0.000 | |
| OTU9 | Bacteroidetes | Bacteroidaceae | Bacteroides | 0.039 | 0.000 | | 0.000 | | 0.000 | | 0.000 | | 0.000 | | 0.000 | | 0.000 | | 0.000 | | 0.003 | | 0.003 | |
| OTU12 | Bacteroidetes | Bacteroidaceae | Bacteroides | 0.012 | 0.000 | | 0.000 | | 0.000 | | 0.000 | | 0.000 | | 0.000 | | 0.000 | | 0.000 | | 0.000 | | 0.003 | |
| OTU117 | Firmicutes | Lactobacillaceae | Lactobacillus | 0.071 | 0.000 | | 0.000 | | 0.000 | | 0.007 | | 0.000 | | 0.003 | | 0.000 | | 0.000 | | 0.000 | | 0.000 | |
| OTU147 | Firmicutes | Lactobacillaceae | Lactobacillus | 0.016 | 0.000 | | 0.000 | | 0.000 | | 0.000 | | 0.000 | | 0.000 | | 0.003 | | 0.000 | | 0.000 | | 0.006 | |
| OTU146 | Firmicutes | Lactobacillaceae | Lactobacillus | 0.024 | 0.000 | | 0.000 | | 0.000 | | 0.000 | | 0.000 | | 0.003 | | 0.000 | | 0.000 | | 0.000 | | 0.000 | |
| OTU91 | Bacteroidetes | Porphyromonadaceae | Parabacteroides | 0.008 | 0.000 | | 0.003 | | 0.000 | | 0.000 | | 0.000 | | 0.000 | | 0.000 | | 0.000 | | 0.000 | | 0.000 | |
| OTU36 | Firmicutes | Lachnospiraceae | Lachnospiracea_incertae_sedis | 0.217 | 0.000 | | 0.000 | | 0.000 | | 0.000 | | 0.000 | | 0.000 | | 0.000 | | 0.000 | | 0.000 | | 0.003 | |
| OTU26 | Firmicutes | Lachnospiraceae | Unclassified | 0.097 | 0.000 | | 0.000 | | 0.000 | | 0.000 | | 0.000 | | 0.000 | | 0.000 | | 0.000 | | 0.000 | | 0.003 | |
| OTU24 | Firmicutes | Lachnospiraceae | Unclassified | 0.058 | 0.000 | | 0.000 | | 0.000 | | 0.000 | | 0.000 | | 0.000 | | 0.000 | | 0.000 | | 0.000 | | 0.003 | |
| OTU159 | Firmicutes | Veillonellaceae | Veillonella | 0.015 | 0.000 | | 0.000 | | 0.000 | | 0.000 | | 0.000 | | 0.000 | | 0.003 | | 0.000 | | 0.000 | | 0.000 | |
| OTU14 | Proteobacteria | Idiomarinaceae | Aliidiomarina | 0.799 | 0.003 | | 0.000 | | 0.000 | | 0.000 | | 0.000 | | 0.003 | | 0.000 | | 0.003 | | 0.003 | | 0.000 | |
| OTU15 | Proteobacteria | Halomonadaceae | Halomonas | 0.521 | 0.003 | | 0.003 | | 0.000 | | 0.003 | | 0.000 | | 0.000 | | 0.006 | | 0.000 | | 0.000 | | 0.000 | |
| OTU18 | Actinobacteria | Dietziaceae | Dietzia | 0.063 | 0.000 | | 0.003 | | 0.000 | | 0.003 | | 0.000 | | 0.003 | | 0.000 | | 0.000 | | 0.000 | | 0.003 | |
| OTU32 | Actinobacteria | Nitriliruptoraceae | Nitriliruptor | 0.016 | 0.000 | | 0.003 | | 0.000 | | 0.000 | | 0.000 | | 0.006 | | 0.000 | | 0.003 | | 0.006 | | 0.000 | |
| OTU16 | Firmicutes | Lachnospiraceae | Fusicatenibacter | 0.081 | 0.000 | | 0.000 | | 0.000 | | 0.003 | | 0.000 | | 0.003 | | 0.000 | | 0.000 | | 0.000 | | 0.000 | |
| OTU22 | Actinobacteria | Nocardioidaceae | Aeromicrobium | 0.039 | 0.000 | | 0.000 | | 0.000 | | 0.000 | | 0.003 | | 0.000 | | 0.000 | | 0.000 | | 0.003 | | 0.000 | |
| OTU88 | Proteobacteria | Phyllobacteriaceae | Unclassified | 0.035 | 0.000 | | 0.000 | | 0.000 | | 0.000 | | 0.000 | | 0.000 | | 0.003 | | 0.009 | | 0.000 | | 0.000 | |
| OTU85 | Proteobacteria | Xanthomonadaceae | Unclassified | 0.031 | 0.000 | | 0.003 | | 0.000 | | 0.000 | | 0.000 | | 0.000 | | 0.000 | | 0.003 | | 0.000 | | 0.000 | |
| OTU23 | Proteobacteria | Enterobacteriaceae | Unclassified | 0.008 | 0.009 | | 0.000 | | 0.000 | | 0.000 | | 0.000 | | 0.003 | | 0.000 | | 0.000 | | 0.000 | | 0.000 | |
| OTU67 | Firmicutes | Ruminococcaceae | Butyricicoccus | 0.012 | 0.000 | | 0.000 | | 0.000 | | 0.000 | | 0.003 | | 0.000 | | 0.000 | | 0.000 | | 0.000 | | 0.000 | |
| OTU164 | Cyanobacteria/Chloroplast | Chloroplast | Streptophyta | 0.004 | 0.000 | | 0.000 | | 0.000 | | 0.000 | | 0.000 | | 0.000 | | 0.000 | | 0.000 | | 0.000 | | 0.003 | |
| OTU94 | Firmicutes | Streptococcaceae | Streptococcus | 0.000 | 0.000 | | 0.000 | | 0.006 | | 0.000 | | 0.000 | | 0.003 | | 0.003 | | 0.000 | | 0.006 | | 0.003 | |
| OTU55 | Actinobacteria | Bifidobacteriaceae | Bifidobacterium | 0.000 | 0.000 | | 0.000 | | 0.003 | | 0.000 | | 0.000 | | 0.000 | | 0.006 | | 0.000 | | 0.000 | | 0.003 | |
| OTU19 | Actinobacteria | Bifidobacteriaceae | Bifidobacterium | 0.000 | 0.000 | | 0.000 | | 0.000 | | 0.000 | | 0.000 | | 0.003 | | 0.003 | | 0.000 | | 0.000 | | 0.000 | |
| OTU130 | Proteobacteria | Rhodocyclaceae | Dechloromonas | 0.000 | 0.000 | | 0.000 | | 0.003 | | 0.000 | | 0.000 | | 0.000 | | 0.006 | | 0.000 | | 0.000 | | 0.000 | |
| OTU83 | Actinobacteria | Bogoriellaceae | Bogoriella | 0.000 | 0.003 | | 0.000 | | 0.000 | | 0.000 | | 0.000 | | 0.003 | | 0.000 | | 0.000 | | 0.000 | | 0.000 | |
| OTU57 | Verrucomicrobia | Verrucomicrobiaceae | Akkermansia | 0.000 | 0.000 | | 0.000 | | 0.000 | | 0.000 | | 0.000 | | 0.000 | | 0.000 | | 0.000 | | 0.000 | | 0.006 | |
| OTU137 | Proteobacteria | Alcanivoracaceae | Alcanivorax | 0.000 | 0.000 | | 0.000 | | 0.000 | | 0.000 | | 0.000 | | 0.000 | | 0.000 | | 0.003 | | 0.000 | | 0.000 | |
| OTU52 | Bacteroidetes | Rikenellaceae | Alistipes | 0.000 | 0.000 | | 0.000 | | 0.000 | | 0.000 | | 0.003 | | 0.000 | | 0.000 | | 0.000 | | 0.000 | | 0.000 | |
| OTU38 | Bacteroidetes | Bacteroidaceae | Bacteroides | 0.000 | 0.000 | | 0.000 | | 0.000 | | 0.000 | | 0.000 | | 0.000 | | 0.000 | | 0.000 | | 0.000 | | 0.003 | |
| OTU75 | Bacteroidetes | Bacteroidaceae | Bacteroides | 0.000 | 0.000 | | 0.000 | | 0.000 | | 0.000 | | 0.000 | | 0.000 | | 0.000 | | 0.000 | | 0.000 | | 0.003 | |
| OTU121 | Actinobacteria | Coriobacteriaceae | Collinsella | 0.000 | 0.000 | | 0.000 | | 0.000 | | 0.000 | | 0.000 | | 0.000 | | 0.000 | | 0.000 | | 0.000 | | 0.003 | |
| OTU45 | Firmicutes | Lachnospiraceae | Coprococcus | 0.000 | 0.000 | | 0.000 | | 0.000 | | 0.000 | | 0.003 | | 0.000 | | 0.000 | | 0.000 | | 0.000 | | 0.000 | |
| OTU86 | Firmicutes | Erysipelotrichaceae | Holdemanella | 0.000 | 0.000 | | 0.000 | | 0.000 | | 0.000 | | 0.000 | | 0.000 | | 0.000 | | 0.000 | | 0.000 | | 0.000 | |
| OTU112 | Bacteroidetes | Porphyromonadaceae | Odoribacter | 0.000 | 0.000 | | 0.000 | | 0.000 | | 0.000 | | 0.000 | | 0.000 | | 0.000 | | 0.000 | | 0.003 | | 0.000 | |
| OTU150 | Bacteroidetes | Porphyromonadaceae | Parabacteroides | 0.000 | 0.000 | | 0.000 | | 0.000 | | 0.000 | | 0.000 | | 0.000 | | 0.000 | | 0.000 | | 0.000 | | 0.003 | |
| OTU34 | Bacteroidetes | Prevotellaceae | Prevotella | 0.000 | 0.000 | | 0.000 | | 0.000 | | 0.000 | | 0.000 | | 0.000 | | 0.000 | | 0.000 | | 0.000 | | 0.003 | |
| OTU154 | Proteobacteria | Rhodocyclaceae | Thauera | 0.000 | 0.000 | | 0.000 | | 0.000 | | 0.000 | | 0.000 | | 0.003 | | 0.000 | | 0.000 | | 0.000 | | 0.000 | |
| OTU59 | Actinobacteria | Microbacteriaceae | Unclassified | 0.000 | 0.000 | | 0.003 | | 0.000 | | 0.000 | | 0.000 | | 0.000 | | 0.000 | | 0.000 | | 0.000 | | 0.000 | |
| OTU129 | Firmicutes | Ruminococcaceae | Unclassified | 0.000 | 0.000 | | 0.000 | | 0.000 | | 0.000 | | 0.000 | | 0.000 | | 0.000 | | 0.000 | | 0.000 | | 0.003 | |

§, Abundance (%) above zero in the mice harboring the OTU is highlighted in gray.
